# Curcumin Can Inhibit Zearalenone-Induced Ferroptosis in Porcine Intestinal Epithelial Cells via the p53/SLC7A11/GPX4 Pathway

**DOI:** 10.3390/toxics13090713

**Published:** 2025-08-24

**Authors:** Dongwei Xiong, Weidong Qi, Miao Long

**Affiliations:** Key Laboratory of Livestock Infectious Diseases, Ministry of Education, College of Animal Science & Veterinary Medicine, Shenyang Agricultural University, Shenyang 110866, China; 2022200183@stu.syau.edu.cn (D.X.); 2022220619@stu.syau.edu.cn (W.Q.)

**Keywords:** zearalenone, curcumin, ferroptosis, lipid peroxidation, IPEC-J2 cells

## Abstract

Zearalenone (ZEA) is a widely distributed estrogenic mycotoxin that can disrupt intestinal barrier integrity by inducing ferroptosis, thereby posing serious risks to animal health. Curcumin (CUR), as a natural polyphenolic compound with multi-target regulatory properties, has attracted increasing attention for its antioxidative and cytoprotective effects; however, its role in ZEA-induced ferroptosis remains poorly understood. In this study, the protective effects of curcumin (CUR) were evaluated in IPEC-J2 cells by co-treating the cells with zearalenone (ZEA) at its LC_50_ (75.23 μM) and curcumin (5 or 15 μM) for 24 h. CCK-8 assays showed that CUR significantly (*p* < 0.05) and highly significantly (*p* < 0.01) improved cell viability in the 5 μM and 15 μM groups, respectively, compared with ZEA alone. CUR co-treatment significantly (*p* < 0.01) restored glutathione (GSH) levels, and markedly (*p* < 0.01) reduced Fe^2+^ accumulation, reactive oxygen species (ROS) production, malondialdehyde (MDA) content, and lipid peroxidation (LPO). Transmission electron microscopy revealed pronounced mitochondrial cristae loss and membrane collapse in ZEA-treated cells, which were visibly alleviated by CUR. At the molecular level, ZEA downregulated GPX4 and SLC7A11 and upregulated ACSL4, FTH1, and p53 (all *p* < 0.01), whereas these changes were significantly reversed (*p* < 0.05 or *p* < 0.01) by CUR. In conclusion, CUR exerts cytoprotective effects against ZEA-induced ferroptosis, likely via modulation of the p53/SLC7A11/GPX4 signaling pathway.

## 1. Introduction

In recent years, feed contamination by mycotoxins has garnered increasing attention, with zearalenone (ZEA), an estrogenic mycotoxin produced by the Fusarium species, being one of the most prevalent contaminants in grains and their by-products [1,2]. Due to its structural similarity to natural estrogens, ZEA exhibits potent estrogenic activity, disrupting the endocrine system in animals by activating estrogen receptors and leading to reproductive disorders, immunosuppression, and hepatic and renal toxicity [3,4]. Especially in livestock and poultry farming, pigs—being highly sensitive species—are particularly vulnerable, with the intestine being the first site of ZEA exposure and playing a key role in its absorption and biotransformation, making it a primary target of ZEA toxicity [5,6]. ZEA exposure can impair intestinal barrier function, and trigger oxidative stress and inflammatory responses, thereby compromising pig health and production performance [7,8]. However, the specific molecular mechanisms by which ZEA induces cellular damage in intestinal epithelial cells remain largely unexplored.

**Figure 1:**
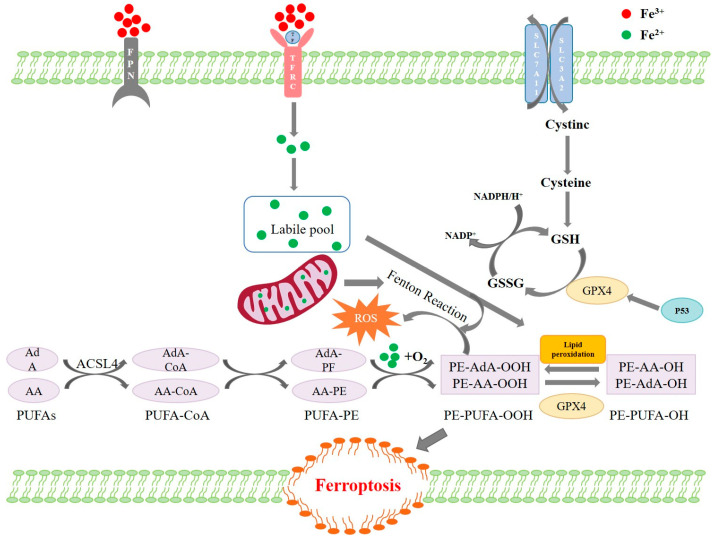
Mechanisms of ferroptosis regulation.

Traditional studies have primarily focused on classical forms of programmed cell death such as apoptosis and necrosis induced by ZEA. In recent years, ferroptosis, a novel iron-dependent form of programmed cell death, has garnered increasing attention due to its pivotal role in various disease models. Ferroptosis is characterized by the accumulation of lipid peroxides on cellular membranes, along with molecular events such as glutathione (GSH) depletion, inactivation of glutathione peroxidase 4 (GPX4), and disruption of intracellular iron homeostasis, ultimately leading to loss of cellular structural integrity [9,10]. Unlike conventional forms of cell death, ferroptosis features distinct metabolic regulatory mechanisms and pharmacological targets. Preliminary evidence indicates that ZEA can induce hallmark features of ferroptosis in various cell types; however, studies in porcine intestinal epithelial cells—a key barrier model—remain limited, particularly regarding systematic elucidation of its regulatory pathways [11]. The occurrence of ferroptosis is tightly regulated by multiple signaling pathways. Among them, the p53/SLC7A11/GPX4 axis is considered a key molecular pathway governing ferroptosis sensitivity [11,12]. As a classical tumor suppressor, p53 can repress the transcriptional activity of SLC7A11 under stress conditions, thereby limiting cystine uptake, impairing GSH synthesis, reducing GPX4 activity, promoting lipid peroxide accumulation, and ultimately inducing ferroptosis [10,13,14]. As shown in Figure 1, studies on the regulatory mechanisms of ferroptosis have primarily focused on GSH metabolism, iron metabolism, and lipid metabolism [15,16]. Thus, this signaling axis plays a central regulatory role in ferroptosis induced by stress-related toxicants. Elucidating whether ZEA mediates ferroptosis in intestinal epithelial cells via this pathway is essential for understanding its toxicological mechanism.

Natural bioactive compounds have emerged as a research hotspot for mitigating ZEA toxicity due to their low toxicity, high efficiency, and multi-targeted properties. Curcumin (CUR), a natural polyphenolic compound extracted from turmeric, exhibits prominent antioxidant and anti-inflammatory activities [17]. Studies have demonstrated that curcumin mitigates oxidative stress by scavenging reactive oxygen species (ROS), enhancing antioxidant enzyme activity, and stabilizing mitochondrial function, thereby exerting anti-ferroptotic effects in models of tumors, neurodegenerative diseases, and renal injury [18,19]. Some studies have shown that curcumin regulates the expression of SLC7A11 and GPX4, thereby influencing GSH levels and modulating ferroptosis [20]. However, whether curcumin confers similar protective effects against ZEA-induced porcine intestinal epithelial cell (IPEC-J2) injury, and whether the underlying mechanism involves the p53/SLC7A11/GPX4 pathway remain to be systematically investigated.

Therefore, this study employed a ZEA-induced IPEC-J2 porcine intestinal epithelial cell model to systematically investigate the protective effects of curcumin and its underlying mechanisms by assessing cell viability, ROS and Fe^2+^ levels, lipid peroxidation, GSH content, and the expression of key regulatory proteins. We aimed to fill this gap by integrating biochemical, morphological, and molecular analyses to determine whether curcumin protects against ZEA-induced ferroptosis in IPEC-J2 cells, and by elucidating the involvement of the p53/SLC7A11/GPX4 signaling pathway. This work not only enriches the molecular understanding of ZEA toxicity but also provides a theoretical basis for the development of natural anti-toxicity intervention strategies.

## 2. Materials and Methods

### 2.1. Culture of IPEC-J2 Cells

Porcine intestinal epithelial cells (IPEC-J2, American Type Culture Collection, Manassas, VA, USA) were maintained in DMEM/F-12 (Procell, Wuhan, China) containing 10% fetal bovine serum (FBS; Hyclone, Marlborough, MA, USA) and 1% penicillin–streptomycin (Procell, Wuhan, China) at 37 °C in a 5% CO_2_ humidified incubator, as previously reported by Cheng et al. [21] and Wang et al. [22]. Cells at passages 10–20 were used for all experiments. When the cultures reached ~80% confluence, cells were dissociated using 0.25% trypsin–EDTA, the reaction was stopped with complete medium, centrifuged, and reseeded at a ratio of 1:3 for further growth.

### 2.2. Cytotoxicity Assay and Relative Cell Viability Measurement

When IPEC-J2 cells reached 80% confluency, they were digested with 0.25% trypsin to prepare a single-cell suspension. After determining the cell concentration using an automated cell counter, the suspension was adjusted to 1 × 10^4^ cells/mL. The well-mixed cell suspension was seeded into 96-well plates at 100 μL per well, ensuring uniform cell distribution across wells (error ≤ ±5%), and preincubated for 24 h at 37 °C in a 5% CO_2_ incubator. After ensuring good cell adherence and 80% confluency, the spent medium was removed, and the cells were subjected to drug treatment.

(1) Curcumin Treatment and Relative Cell Viability Assay (CCK-8 assay)

To evaluate the effect of CUR (purity > 98%, Solarbio, Beijing, China) on the viability of IPEC-J2 cells, a CUR concentration gradient of 0, 5, 10, 15, 20, 25, and 30 μmol/L was established. Following the method described by Wang et al. [23], CUR was first dissolved in dimethyl sulfoxide (DMSO) to prepare a 60 mM stock solution, stored at −20 °C in the dark, and freshly diluted in DMEM/F-12 medium to the target concentrations immediately before use. The same volume of DMSO was added to the control group to ensure that the final DMSO concentration in all treatments, including controls, was less than 0.1% (*v*/*v*). Each treatment included six biological replicates. Cells were incubated with CUR for 24 h in the dark, followed by the addition of 10 μL of CCK-8 reagent to each well and further incubation for 2 h. The absorbance (OD) at 480 nm was measured using a microplate reader to determine relative cell viability.

(2) Zearalenone Treatment and Relative Cell Viability Assay (CCK-8 assay)

ZEA (purity > 99%, Pribolab, Qingdao, China) treatment concentrations were set at 50, 60, 70, 80, 90, 100, and 110 μmol/L. The experiment included ZEA-treated and control groups, following the same incubation conditions and detection procedures as in the CUR experiment. After 24 h of ZEA exposure, CCK-8 reagent (Solarbio, Beijing, China) was added, and absorbance was measured at 480 nm to determine OD values.

(3) Investigation of the Combined Effects of CUR and ZEA (CCK-8 assay)

To investigate the combined effects of CUR and ZEA, co-treatment experiments were designed based on the median lethal concentration (LC_50_) of ZEA. CUR concentrations ranged from 5 to 30 μmol/L, while ZEA was fixed at its LC_50_ dose (75 μmol/L). The experiment included control, ZEA (75 μmol/L), ZEA + CL (75 μmol/L ZEA + 5 μmol/L CUR), and ZEA + CH (75 μmol/L ZEA + 15 μmol/L CUR), with six biological replicates in each group. The exposure time was set to 24 h, and relative cell viability following combined treatment was assessed using the CCK-8 assay, employing the same protocol as for the single-treatment groups.

### 2.3. Measurement of GSH, Fe^2+^, LPO, and MDA Contents

When IPEC-J2 cells reached 80% confluency, four groups were established: control, ZEA (75 μmol/L), ZEA + CL (75 μmol/L ZEA + 5 μmol/L CUR), and ZEA + CH (75 μmol/L ZEA + 15 μmol/L CUR), with three biological replicates per group. Following 24 h of exposure, the medium was removed, cells were rinsed twice with PBS, the residual liquid was completely aspirated, and the cells were collected using a cell scraper. A cell suspension was prepared in 2 mL PBS and subjected to cell counting. The method is described by Sun et al. [24].

(1) Measurement of GSH Contents

During sample preparation, approximately 1 × 10^6^ cells were resuspended in 300–500 μL of PBS and disrupted using ultrasonic homogenization. The resulting lysate was centrifuged at 10,000 rpm for 10 min at 4 °C, and the supernatant was retained for further analysis. Simultaneously, protein levels were quantified via the Bicinchoninic acid (BCA) method (Solarbio, Beijing, China). Briefly, 20 μL of each sample or BSA standard (0.0 μg/mL, 5.0 μg/mL, 10.0 μg/mL, 15.0 μg/mL, 20.0 μg/mL, 30.0 μg/mL, 40.0 μg/mL, 50.0 μg/mL) was pipetted into a 96-well plate, mixed with 200 μL of BCA working reagent, and incubated at 37 °C for 30 min. Absorbance at 562 nm was then recorded, and protein concentrations were determined based on the generated standard curve. GSH content was measured according to the instructions of the GSH assay kit (Njjcbio, Nanjing, China). The sample supernatant was mixed with an equal volume of Reagent I and centrifuged at 4500× *g* for 10 min, and the supernatant was collected again. In the detection system, 25 μL of Reagent III was first added to the 96-well plate, followed by 100 μL of Reagent I, pre-prepared GSH standard solution or sample supernatant, and finally 100 μL of Reagent II. After mixing, the reaction was allowed to proceed for 5 min, and absorbance at 405 nm was measured with a microplate reader. Data analysis was performed using a standard curve fitted with the equation y = ax + b, and the GSH content (μmol/g protein) was calculated as follows:

GSH = ((ΔA − b)/a) × 2/C _pr_ (1)

where ΔA represents the absorbance difference between the sample and blank, a is the slope of the standard curve, b is the intercept, and C _pr_ is the sample protein concentration (g/L).

(2) Measurement of Fe^2+^ Contents

The determination of intracellular Fe^2+^ ion content was carried out using a commercial assay kit (Njjcbio, Nanjing, China) in accordance with the supplied protocol. Briefly, harvested cells (1 × 10^6^) were combined with 0.2 mL of Reagent I, mixed, and subjected to ice-cold lysis for 10 min. The lysates were centrifuged at 15,000× *g* for 10 min at 4 °C, after which the clarified supernatants were collected for measurement. Quantification was performed using a colorimetric approach. A 96-well plate was prepared with standard, test, and control wells. For standards, 80 μL of Fe^2+^ ion standard solutions at different concentrations was pipetted into the wells; for the test and control groups, 80 μL of the sample was added. Reagent II (80 μL) was dispensed into the control wells, while Reagent III (80 μL) was added to both test and control wells. Following gentle mixing, the plate was incubated at 37 °C for 10 min before reading. Following termination of the reaction, optical density (OD) was measured at 593 nm. The relationship between standard concentration and OD is described by the linear equation y = ax + b, where y represents the OD of standards minus the blank OD (at zero concentration), and x represents the concentration of the standard. The formula for calculating Fe^2+^ content (nmol/10^6^ cells) is as follows:

Fe^2+^ = ((A _test_ − A _control_
− b)/a) × 10^6^/N × 1/V (2)

where A _test_ and A _control_ are the absorbance values of the test and control wells, respectively; a is the slope of the standard curve; b is the intercept; N is the number of cells in the sample; and V is the lysis volume (mL).

(3) Measurement of Lipid peroxidation (LPO) Contents

For sample processing, 1 × 10^6^ cells were resuspended in 500 μL PBS and subjected to ultrasonic homogenization. The homogenized suspension was centrifuged at 10,000 rpm for 10 min at 4 °C, and the resulting supernatant was harvested for downstream analysis. LPO quantification was performed with a LPO ELISA detection kit (Njjcbio, Nanjing, China) following the provided protocol. A 96-well microplate was prepared with standard, sample, and blank wells. Standard wells received 50 μL of LPO standards at different concentrations. For sample wells, 10 μL of sample was dispensed, followed by 40 μL dilution buffer and mixing. Blank wells contained no solution and were used as background controls. Subsequently, 100 μL of HRP-labeled primary antibody working solution was dispensed into the standard and sample wells. After sealing the microplate, it was incubated at 37 °C for 60 min. Upon completion of incubation, the reaction fluid was removed, and the plate was blotted dry with absorbent paper. Each well was washed five times with 350 μL phosphate buffer, allowing for a 60 s soak per wash to ensure thorough rinsing. During the chromogenic reaction, 50 μL of a substrate mixture (solution A and B) was added to each well and incubated at 37 °C in the dark for 15 min. To terminate the reaction, 50 μL of stop solution was added to each well, and absorbance at 450 nm was measured immediately using a microplate reader.

(4) Measurement of Malondialdehyde (MDA) Contents

For sample preparation, 1 × 10^6^ cells were resuspended in 500 μL PBS and subjected to ultrasonic homogenization. The obtained homogenate was used for downstream measurements. The quantification of MDA was performed following the MDA detection kit (Njjcbio, Nanjing, China) protocol. Protein concentrations were measured by the BCA method, consistent with the approach used in GSH analysis. Three groups were established: blank, standard, and experimental groups. In the blank group, 0.1 mL of anhydrous ethanol was added; in the standard group, 0.1 mL of 10 nmol/mL standard solution was used; and in the experimental group, 0.1 mL of test sample was placed into a 1.5 mL centrifuge tube. After adding suitable volumes of working solution to each group and mixing thoroughly, the tubes were sealed and incubated in a 100 °C water bath for 40 min. After heating, the samples were cooled under running water to room temperature and centrifuged at 1078× *g* for 10 min. An aliquot (0.25 mL) of the supernatant was transferred to a microplate, and optical density (OD) was recorded at 532 nm. Lipid peroxidation end-product levels (nmol/mg protein) were calculated as follows:

MDA = (A _sample_ − A _blank_)/(A _control_ − A _blank_) × 10/C _pr_ (3)

where A indicates the absorbance values for each group, 10 denotes the standard concentration (nmol/mL), and C _pr_ refers to the sample protein concentration (mg/mL).

### 2.4. Flow Cytometric Analysis of ROS Production in IPEC-J2 Cells

At 80% confluency, IPEC-J2 cells were divided into four groups: control, ZEA, ZEA + CL, and ZEA + CH, with three biological replicates per group. Prior to ROS analysis, the culture medium was discarded, cells were washed twice with PBS to remove residual substances, collected with a cell scraper, resuspended in 2 mL PBS, and counted to prepare for ROS level assessment.

Cells from each treatment group were rinsed twice with PBS, digested using 0.25% trypsin, and centrifuged at 1000 rpm for 5 min to obtain the cell pellets. Cell density was standardized to 1 × 10^6^ cells. Reactive oxygen species (ROS) levels were evaluated employing a 2′,7′-dichlorodihydrofluorescein diacetate (DCFH-DA) fluorescence assay kit (Servicebio, Beijing, China). The probe solution was prepared by diluting DCFH-DA 1:1000 in serum-free DMEM to achieve a working concentration of 10 μmol/L. One milliliter of the probe-containing medium was added to each cell sample, followed by thorough resuspension and incubation at 37 °C for 20 min in the dark, with intermittent gentle agitation to promote dye uptake. After staining, cells were washed three times with serum-free DMEM to remove unbound probe, and then resuspended in 500 μL serum-free DMEM for subsequent ROS analysis. Fluorescence was measured using a Sony SH800 flow cytometer (San Jose, CA, USA) with settings of 488 nm excitation and 525 nm emission. To minimize artifacts caused by oxidative stress, the interval between probe loading and fluorescence detection was kept as short as possible, ensuring accurate and reproducible measurement of ROS levels.

### 2.5. Observation of Mitochondrial Damage in IPEC-J2 Cells by Transmission Electron Microscopy

IPEC-J2 cells were cultured in T75 flasks, and when the cells reached approximately 80% confluency with good growth status, mitochondrial structure observation experiments were initiated. Grouping and drug treatments followed the protocols described in Section 2.3 for ZEA and CUR exposure, with a treatment duration of 24 h. After exposure, the culture medium was discarded, and the cells were washed twice with 9 mL PBS buffer to completely remove the residual medium. Next, 3 mL of 0.25% trypsin was added for cell detachment, and the reaction was terminated with 3 mL of the complete medium. The collected cells were transferred to centrifuge tubes and spun at 1000 rpm for 5 min. The supernatant was discarded, and the cell pellet was retained. At this stage, the cell mass was approximately the size of a mung bean. An equal volume of 2.5% glutaraldehyde fixative (room temperature, pH 7.2–7.4) was slowly added to the pellet, and gentle pipetting was used to fully resuspend the cells. Fixation was carried out at room temperature in the dark for 30 h. After fixation, samples were stored at 4 °C and later examined using transmission electron microscopy (Hitachi, H-7000, Tokyo, Japan) for image acquisition and analysis of mitochondrial morphological changes and damage features among different groups.

### 2.6. Validation of Target Gene Expression

When IPEC-J2 cells reached roughly 80% confluence, they were allocated into four experimental sets: control, ZEA, ZEA + CL, and ZEA + CH, each containing three independent replicates. Treatments were applied for 24 h, after which the culture medium was removed, and cells were rinsed twice with PBS. The harvested cells were resuspended in PBS and counted. Total RNA was isolated using the Trizol reagent (Takara, Kyoto, Japan) according to standard procedures, including chloroform extraction, precipitation with isopropanol, and subsequent washing in 75% ethanol. The RNA pellet was reconstituted in RNase-free water. RNA yield and purity were assessed via a micro-spectrophotometer, with only samples showing an A260/A280 ratio of 1.8–2.1 processed for reverse transcription. cDNA was generated by eliminating genomic DNA (42 °C, 2 min), followed by first-strand synthesis (37 °C, 15 min; 85 °C, 5 s). The synthesized cDNA was either used immediately for qPCR analysis or preserved at −80 °C for later use.

Quantitative PCR was carried out to measure the transcription levels of ferroptosis-associated genes, namely, p53, SLC7A11, GPX4, ACSL4, and FTH1. Primer sequences were designed from NCBI database entries and verified using the Primer-BLAST tool (Table 1). Each 10 μL reaction consisted of 5 μL of 2× Synergy Brands (SYBR) Green Master Mix (Vazyme, Nanjing, China), 0.2 μL of each primer, 1 μL of cDNA template, and nuclease-free water to reach the final volume. The thermal cycling program began with denaturation at 95 °C for 30 s, followed by 40 amplification cycles (95 °C for 10 s, 60 °C for 30 s), and was concluded with a melting curve analysis. The relative expression of target genes was calculated using the 2^−ΔΔCt^ approach, where ΔΔCt = (Ct_target − Ct_reference)_treatment − (Ct_target − Ct_reference)_control, with Glyceraldehyde-3-Phosphate Dehydrogenase(GAPDH) serving as the internal normalization control to ensure data reliability.

### 2.7. Western Blotting to Assess the Expression of Ferroptosis-Associated Proteins

IPEC-J2 cells were cultured to approximately 80% confluency and allocated into four groups: control, ZEA, ZEA + CL, and ZEA + CH, each with three biological replicates. After 24 h of exposure, the medium was discarded, and the cells were rinsed twice with PBS, collected using a cell scraper, resuspended in PBS, and counted. The lysis buffer was composed of radio immunoprecipitation assay (RIPA) buffer, protease inhibitor, and phenyl methyl sulfonyl fluoride (PMSF) (Solarbio, Beijing, China) in a 100:1:1 ratio. Following 30 min of lysis, samples were centrifuged at 14,000× *g* for 30 min at 4 °C, and the supernatant was harvested as total protein. Concentrations were quantified using the BCA method, followed by mixing with 5× sample buffer. Proteins were denatured at 100 °C for 10 min, cooled on ice, and stored at –80 °C until use. SDS-PAGE was carried out with gels of appropriate acrylamide concentration. The voltage was set to 80 V for stacking, then increased to 120 V once the samples entered the separating gel and maintained until completion. Protein transfer was performed using activated polyvinylidene difluoride (PVDF) membranes and pre-cooled transfer buffer, under wet transfer conditions at a constant current of 400 mA. Following transfer, membranes were blocked with 5% non-fat milk in TBST at 4 °C for 2 h, washed five times in tris-buffered saline with tween-20 (TBST), and incubated for 2 h with primary antibodies against p53, SLC7A11, GPX4, ACSL4, and FTH1 (Abclone, Wuhan, China). Membranes were then incubated with secondary antibodies for 2 h, washed, and developed using enhanced chemiluminescence (ECL) (Solarbio, Beijing, China). Bands were visualized and recorded using a gel imaging system, and relative expression levels were quantified.

## 3. Results

### 3.1. The Cytotoxic Effects of ZEA and CUR on IPEC-J2 Cells

**Figure 2:**
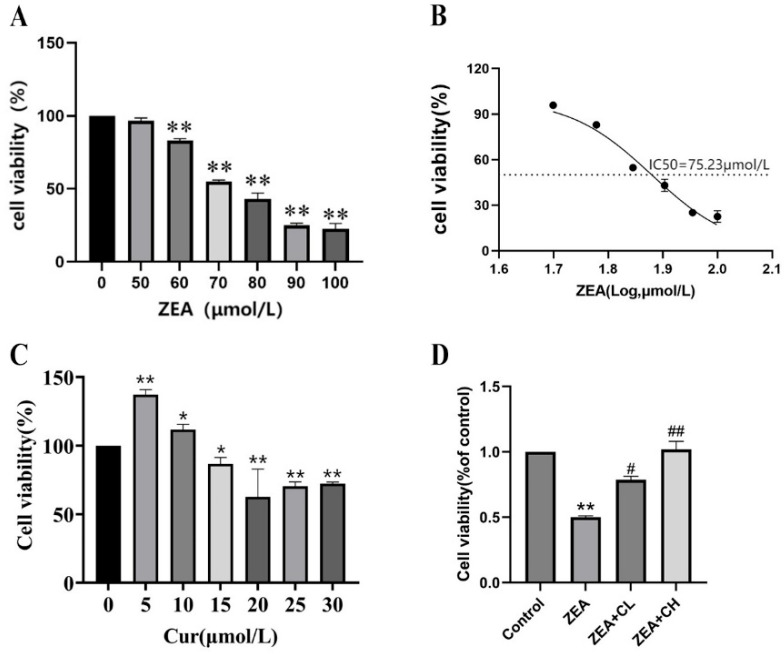
Protective role of curcumin against zearalenone-induced cytotoxicity in IPEC-J2 cells: (**A**) effect of different concentrations of ZEA on the relative viability of IPEC-J2 cells. (**B**) Dose–response sigmoidal curve of ZEA-induced cytotoxicity in IPEC-J2 cells. (**C**) Effect of different concentrations of CUR action on the relative survival of IPEC-J2 cells. (**D**) Effect of CUR on the relative survival rate of IPEC-J2 cells. “*” denotes a statistically significant difference versus the blank control group (*p* < 0.05), while “**” denotes a highly significant difference (*p* < 0.01). “#” signifies a significant difference versus the ZEA group (*p* < 0.05), and “##” indicates a highly significant difference (*p* < 0.01).

The CCK-8 assay was used to evaluate the effects of different concentrations of ZEA on the viability of IPEC-J2 cells to determine its individual and combined effects on cell survival. As shown in Figure 2A, ZEA exposure led to a dose-dependent decrease in cell viability, with a significant reduction observed starting at 60 μmol/L (*p* < 0.01). The calculated IC50 of ZEA was 75.23 μmol/L (Figure 2B), and this concentration was selected for further investigation of ZEA-induced cytotoxicity. These results highlight the potent cytotoxicity of ZEA at concentrations above 60 μmol/L, indicating that even relatively low doses can cause significant cellular damage. In contrast, CUR exhibited a concentration-dependent enhancement in cell viability within the lower concentration range. As shown in Figure 2C, CUR significantly promoted cell survival at concentrations between 5 μmol/L and 10 μmol/L, with the highest viability observed at 10 μmol/L. However, CUR treatment at concentrations of 15 μmol/L or higher resulted in a significant decline in cell viability, indicating potential cytotoxicity at elevated doses. These results suggest a biphasic effect of CUR, where low-to-moderate concentrations promote cell survival, whereas excessive doses may trigger cellular stress or toxicity.

To investigate whether curcumin (CUR) could attenuate zearalenone (ZEA)-induced cytotoxicity, we examined the combined effects of ZEA and CUR on the viability of IPEC-J2 cells. As shown in Figure 2D, treatment with ZEA alone significantly reduced cell viability compared to the control group (*p* < 0.01). However, co-treatment with low-dose CUR (5 μmol/L) significantly restored cell survival (*p* < 0.05). Importantly, high-dose CUR (15 μmol/L) exerted an even stronger protective effect, markedly reversing ZEA-induced cytotoxicity (*p* < 0.01). These findings indicate that both low and high concentrations of CUR effectively counteract ZEA-induced cytotoxicity. The observed dose-dependent response suggests that CUR provides optimal protection at moderate concentrations, while excessive doses may compromise viability due to pro-oxidative or cytotoxic effects.

### 3.2. The Impact of ZEA and CUR on Oxidative Stress and Iron Homeostasis in IPEC-J2 Cells

**Figure 3:**
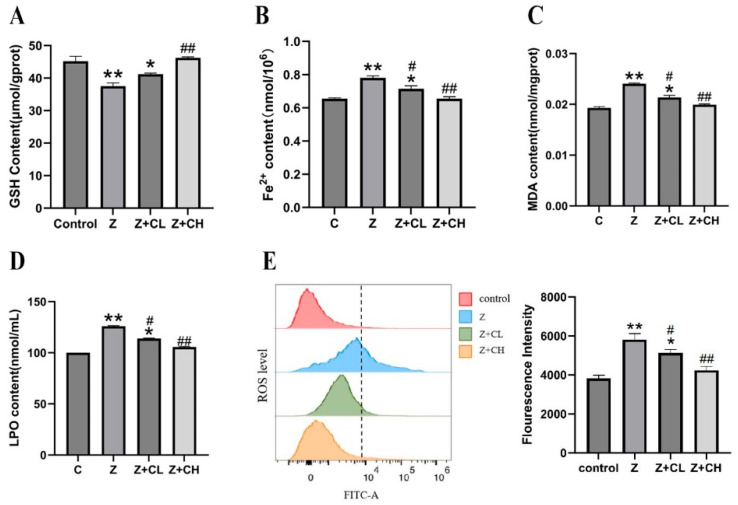
CUR mitigates ZEA-induced oxidative injury by restoring antioxidant capacity and iron homeostasis in IPEC-J2 cells: (**A**) Effects of various drug treatments on the GSH content in IPEC-J2 Cells. (**B**) Effect of different drug treatments on Fe^2+^ content in IPEC-J2 cells. (**C**,**D**) Impact of ZEA exposure on the oxidative system in IPEC-J2 cells. (**E**) Effect of different drug treatments on ROS content in IPEC-J2 cells. “*” denotes a statistically significant difference versus the blank control group (*p* < 0.05), while “**” denotes a highly significant difference (*p* < 0.01). “#” signifies a significant difference versus the ZEA group (*p* < 0.05), and “##” indicates a highly significant difference (*p* < 0.01).

To investigate the effects of ZEA and CUR on oxidative stress and its underlying mechanisms in IPEC-J2 cells, we measured key biomarkers including glutathione (GSH), Fe^2+^, and lipid peroxidation markers. Initially, the influence of ZEA and CUR on intracellular GSH levels in IPEC-J2 cells was assessed. According to Figure 3A, treatment with 75 μmol/L ZEA markedly decreased GSH levels relative to controls (*p* < 0.01). Administration of a low CUR concentration (5 μmol/L) significantly ameliorated GSH depletion (*p* < 0.05), while a high concentration (15 μmol/L) produced a more substantial recovery (*p* < 0.01). These results indicate that CUR protects against ZEA-induced GSH consumption and contributes to the recovery of antioxidant defense in IPEC-J2 cells.

Subsequently, the effects of ZEA and CUR on intracellular Fe^2+^ levels were assessed. Figure 3B illustrates that exposure to 75 μmol/L ZEA markedly elevated intracellular Fe^2+^ levels (*p* < 0.01), suggesting an imbalance in iron homeostasis caused by ZEA. In contrast, administration of both low and high concentrations of CUR significantly decreased Fe^2+^ levels, with the high concentration exerting the strongest effect (*p* < 0.01). Collectively, these results indicate that CUR can modulate iron homeostasis efficiently, potentially helping to prevent ferroptosis linked to ZEA-induced oxidative injury.

Additionally, ZEA- and CUR-induced alterations in the oxidative system were assessed by quantifying lipid peroxidation products (LPO, MDA) and reactive oxygen species (ROS) levels. Figure 3C illustrates that ZEA treatment markedly elevated LPO and MDA levels (*p* < 0.01), suggesting an increase in lipid peroxidation. Likewise, Figure 3D demonstrates a significant increase in ROS levels in cells exposed to ZEA (*p* < 0.05). Administration of CUR at 5 μmol/L significantly decreased LPO and MDA levels (*p* < 0.05), whereas 15 μmol/L CUR led to a more substantial decline (*p* < 0.01). Furthermore, CUR administration lowered ROS levels in a dose-dependent fashion, and high-dose CUR exhibited a highly significant reduction (*p* < 0.01) (Figure 2B). Collectively, these results demonstrate that CUR at both low and high concentrations exhibits potent antioxidant effects, alleviating ZEA-induced oxidative injury and efficiently suppressing oxidative-stress-related lipid peroxidation and ROS production.

### 3.3. The Impact of ZEA and CUR on Mitochondrial Injury in IPEC-J2 Cells

**Figure 4:**
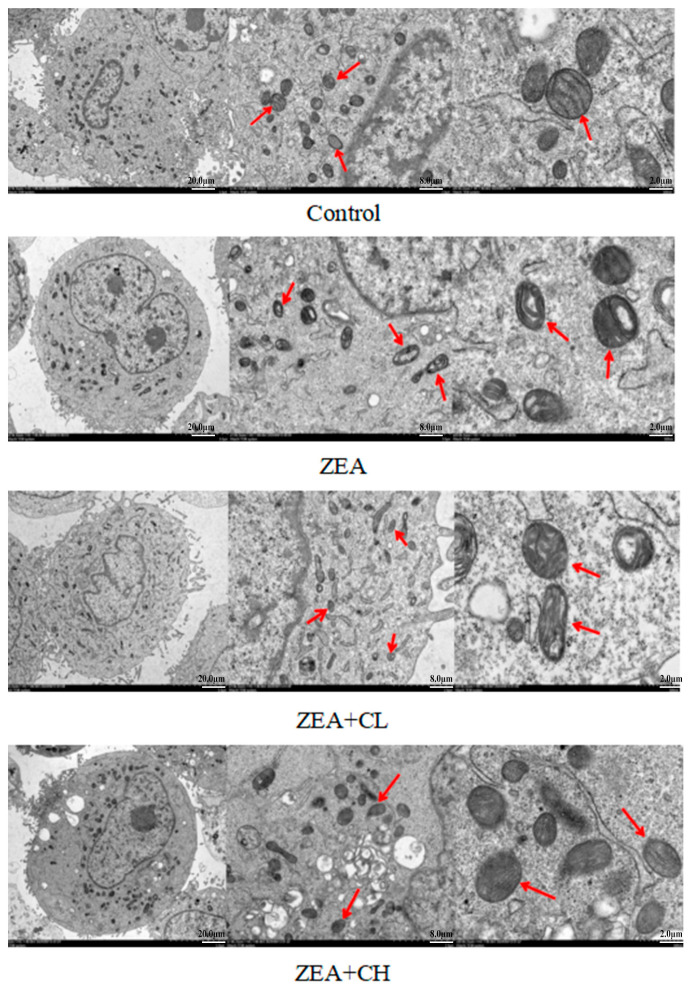
Alterations in the mitochondrial structure of IPEC-J2 cells following treatment with various agents: red arrows highlight alterations in the mitochondrial structure within the cytoplasm of IPEC-J2 cells; the magnifications of the rectangular insets for each group are 2.5K×, 7K×, and 20K×, respectively.

**Figure 5:**
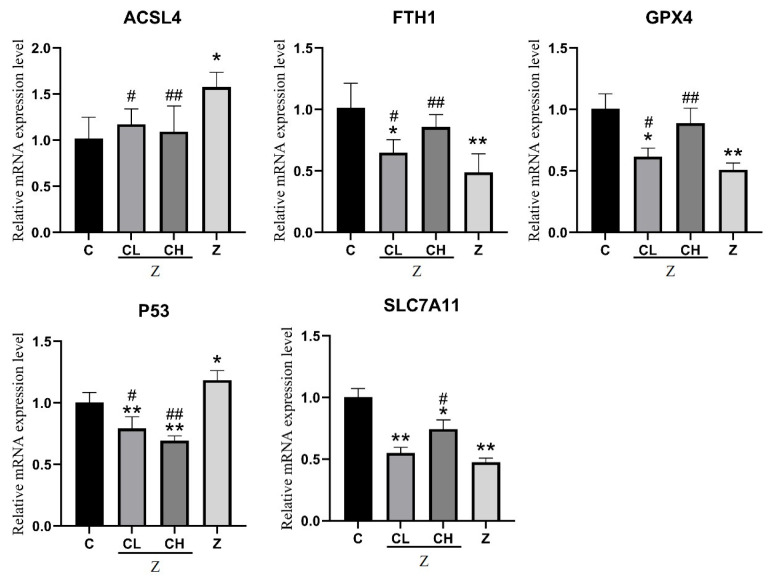
Alterations in ferroptosis-associated gene mRNA expression in IPEC-J2 cells following treatment with varying CUR concentrations: “*” denotes a statistically significant difference versus the blank control group (*p* < 0.05), while “**” denotes a highly significant difference (*p* < 0.01). “#” signifies a significant difference versus the ZEA group (*p* < 0.05), and “##” indicates a highly significant difference (*p* < 0.01).

Mitochondria play a critical role in cellular energy production and maintenance of homeostasis; therefore, we further investigated the effects of ZEA and CUR on mitochondrial integrity and function in IPEC-J2 cells. Figure 3, Figure 4 and Figure 5 demonstrate that treatment of IPEC-J2 cells with 75 μmol/L ZEA induced pronounced mitochondrial morphological changes, such as swelling, cristae disintegration, and compromised membrane integrity. These features suggest mitochondrial dysfunction, a condition frequently linked to oxidative stress and subsequent cell damage. Disruption of mitochondrial morphology compromises cellular energy production and may initiate apoptosis, underscoring the potential cytotoxic effects of ZEA on IPEC-J2 cells. Conversely, CUR treatment displayed protective effects on mitochondrial structure and function in cells exposed to ZEA. Administration of a high CUR concentration (15 μmol/L) significantly alleviated ZEA-induced mitochondrial injury. Restoration of cristae, improved structural clarity, reduced swelling, and enhanced membrane integrity were observed. Even low-dose CUR (5 μmol/L) partially rescued mitochondrial morphology, as evidenced by decreased swelling and improved structural integrity relative to the ZEA-only treatment.

### 3.4. mRNA Expression Levels of Ferroptosis-Related Genes in IPEC-J2 Cells

We examined the mRNA expression profiles of pivotal ferroptosis-associated genes in IPEC-J2 cells following ZEA exposure and CUR intervention. The investigated genes—ACSL4, p53, FTH1, SLC7A11, and GPX4—are recognized for their essential roles in modulating ferroptosis, maintaining iron homeostasis, and mediating oxidative stress responses. Figure 5 illustrates that treatment with 75 μmol/L ZEA markedly altered the mRNA expression levels of ferroptosis-associated genes in IPEC-J2 cells. More specifically, the ACSL4 (*p* < 0.01) and p53 (*p* < 0.05) expression levels were significantly elevated, while those of FTH1 (*p* < 0.01), SLC7A11 (*p* < 0.01), and GPX4 (*p* < 0.01) were notably reduced relative to controls. These observations suggest that ZEA exposure triggers ferroptosis in IPEC-J2 cells, as evidenced by increased expression of ACSL4 and p53 (which drive lipid peroxidation and iron deposition) and decreased levels of protective genes FTH1, SLC7A11, and GPX4. CUR administration significantly reversed ZEA-induced alterations in ferroptosis-associated gene expression. In comparison with the ZEA group, low-dose curcumin (5 μmol/L) significantly increased FTH1, GPX4, and p53 levels (*p* < 0.05) and reduced ACSL4 expression (*p* < 0.01). A higher dose of curcumin (15 μmol/L) further boosted FTH1 and GPX4 expression and markedly suppressed ACSL4 and p53 levels (*p* < 0.01), indicating a dose-dependent protective effect against ferroptosis through regulation of genes involved in lipid peroxidation, iron balance, and antioxidant defense.

### 3.5. Expression Levels of Ferroptosis-Related Proteins in IPEC-J2 Cells

**Figure 6:**
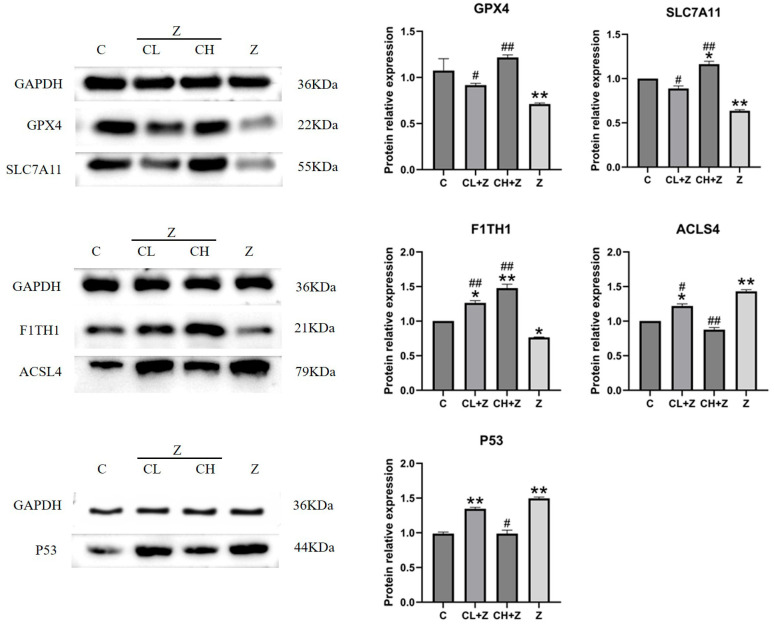
Alterations in ferroptosis-associated protein expression levels in IPEC-J2 cells following treatment with varying CUR concentrations: “*” denotes a statistically significant difference versus the blank control group (*p* < 0.05), while “**” denotes a highly significant difference (*p* < 0.01). “#” signifies a significant difference versus the ZEA group (*p* < 0.05), and “##” indicates a highly significant difference (*p* < 0.01).

We subsequently examined the expression of pivotal ferroptosis-associated proteins in IPEC-J2 cells following ZEA exposure and CUR intervention. Figure 6 demonstrates that exposure to 75 μmol/L ZEA led to pronounced changes in ferroptosis-associated protein expression: FTH1 (*p* < 0.01), GPX4 (*p* < 0.01), and SLC7A11 (*p* < 0.01) levels were significantly reduced, whereas ACSL4 (*p* < 0.05) and p53 (*p* < 0.01) levels were markedly increased. CUR treatment effectively reversed ZEA-induced changes in the expression of multiple ferroptosis-associated proteins. Relative to the ZEA group, low-dose CUR (5 μmol/L) significantly increased FTH1 and GPX4 expression (*p* < 0.05), and substantially reduced ACSL4 (*p* < 0.01) and p53 (*p* < 0.05) levels. Administration of high-dose CUR (15 μmol/L) led to even greater increases in FTH1 and GPX4 expression and a more marked suppression of ACSL4 and p53 levels (*p* < 0.01). CUR confers protection against ferroptosis through modulation of ferroptosis-associated protein expression. Upregulation of FTH1 and GPX4 by CUR contributes to re-establishing iron balance and bolstering antioxidant defenses, both of which are critical for ferroptosis prevention. Additionally, suppression of ACSL4 and p53 by CUR may mitigate lipid peroxidation and restrain the activation of ferroptosis-associated cell death pathways.

## 4. Discussion

Ferroptosis, defined as a programmed cell death modality marked by iron-dependent lipid peroxidation, has emerged as a focal point in toxicology research in recent years. While some studies have indicated the potential of certain mycotoxins to trigger ferroptosis, the underlying molecular mechanisms are still not fully understood. Zearalenone (ZEA), a mycotoxin with widespread occurrence, has been closely linked to damage of the intestinal epithelium in animals [25]. The present study seeks to elucidate the mechanisms by which ZEA induces ferroptosis in IPEC-J2 cells and to assess the protective efficacy of curcumin (CUR) in counteracting ZEA-induced ferroptosis. Expression analyses of ferroptosis-associated genes and proteins in IPEC-J2 cells co-treated with ZEA and CUR revealed that ZEA induces ferroptosis via perturbation of iron homeostasis and oxidative stress, whereas CUR exerts protective effects through regulation of these critical targets.

This study demonstrated that ZEA exposure significantly reduced the viability of IPEC-J2 cells, indicating its pronounced cytotoxic effects. CCK-8 assay results showed that ZEA exerted a strong dose-dependent cytotoxicity at concentrations exceeding 75 μmol/L. In subsequent ferroptosis assays on IPEC-J2 cells, we found that ZEA increased intracellular markers such as Fe^2+^, ROS, LPO, and MDA, thereby exacerbating oxidative stress and inducing ferroptosis. These findings are consistent with previous studies showing that ZEA disrupts cellular iron homeostasis, increases free iron accumulation, activates Fenton reactions in the redox cycle, and produces ROS, which, in turn, causes lipid peroxidation damage [26,27,28]. Moreover, previous studies have shown that ROS accumulation attacks polyunsaturated fatty acids in cell membranes, leading to membrane structural damage and ultimately cell death; this was further corroborated in our study [29]. As ZEA-induced oxidative damage is closely linked to iron homeostasis imbalance, this serves as a critical prerequisite for ferroptosis; thus, the toxicological effects of ZEA-induced ferroptosis are supported by multiple molecular markers in our study.

ZEA induces oxidative stress and lipid peroxidation and further engages in modulating cell death by regulating the expression of critical molecules within multiple ferroptosis signaling pathways. In this study, ZEA exposure resulted in significant changes in the expression of ferroptosis-related genes and proteins in IPEC-J2 cells. The upregulation of ACSL4 and p53, along with the downregulation of FTH1, SLC7A11, and GPX4, indicates that ZEA induced ferroptosis. ACSL4, a key molecular marker of ferroptosis regulation, specifically modifies arachidonic acid via its encoded acyl-CoA synthetase, thereby altering the lipid composition of cell membranes and influencing sensitivity to ferroptosis [30,31]. Intracellular iron is mainly stored in ferritin; during ferritinophagy, Fe^2+^ is released, leading to increased labile iron pool (LIP) concentrations and triggering cytotoxic effects [32,33]. Previous studies have shown that the ferritin heavy-chain subunit FTH1 effectively regulates Fe^2+^ release by rapidly oxidizing Fe^2+^ to Fe^3+^ and incorporating it into the ferritin shell, thereby exerting cytoprotective effects [34,35,36]. A reduction in FTH1 expression implies intracellular free iron accumulation, which exposes cells to heightened levels of iron-dependent oxidative stress. In summary, this study indicates that ZEA disrupts iron homeostasis through multiple mechanisms, exacerbates iron-dependent cellular injury, and promotes ferroptosis.

Concurrently, p53 upregulation is tightly linked to the activation of oxidative stress within cells. By suppressing SLC7A11 expression, p53 reduces intracellular antioxidant synthesis, thereby diminishing cellular antioxidant defenses and heightening susceptibility to ferroptosis [37,38]. Studies have shown that T-2 toxin suppresses SLC7A11 expression [39]. Moreover, in cells deficient in SLC7A11, the extent of ferroptosis was not aggravated, whereas overexpression of SLC7A11 significantly alleviated the ferroptosis enhancement caused by T-2 toxin [40]. SLC7A11, as a key signaling protein in ferroptosis, functions primarily by transporting cystine into cells in an ATP-dependent manner, thereby maintaining intracellular GSH synthesis [41,42]. GSH serves as the essential reducing substrate for GPX4, which protects cells from oxidative damage by catalyzing the reduction in lipid peroxides [43]. The expression level of SLC7A11 directly determines GSH concentration, which, in turn, affects GPX4 activity and cellular resistance to oxidative damage [42]. In the study by Song et al., GPX4 was found to catalyze the conversion of GSH to GSSG during redox reactions and simultaneously eliminate peroxides and hydroxyl radicals generated from cellular respiration and metabolism [44]. This process stabilizes cell membranes and preserves physiological functions, thereby preventing peroxidative damage and inhibiting ferroptosis. A similar phenomenon was observed by Xu et al., where restriction of SLC7A11 function reduced GSH synthesis, impaired GPX4 activity and expression, and consequently led to ferroptosis [45]. Based on these findings, Tao et al. confirmed that p53 represses SLC7A11 expression, thereby inhibiting cystine transport via the System Xc^−^ antiporter [46]. This repression lowers GPX4 enzymatic activity, reduces cellular antioxidant capacity, promotes ROS accumulation, and ultimately triggers ferroptosis [47]. Therefore, our study suggests that ZEA exerts a significant inhibitory effect on the SLC7A11-GSH-GPX4 antioxidant axis, and such systemic suppression of the antioxidant pathway represents a key molecular mechanism underlying its induction of ferroptosis.

CUR, a natural antioxidant, has been extensively studied and applied in the treatment of various oxidative-stress-related diseases [48,49]. To assess the protective role of CUR against ZEA-induced cellular injury, we supplemented different concentrations of CUR to the ZEA-exposed group and compared their effects. The results showed that CUR significantly improved the viability of IPEC-J2 cells and reduced the accumulation of ROS, LPO, and MDA induced by ZEA. More importantly, CUR significantly restored the function of the intracellular antioxidant defense system, particularly by recovering FTH1 and GPX4 expression and upregulating SLC7A11 levels, thereby alleviating ZEA-induced ferroptosis. It is noteworthy that, while a high concentration of CUR (15 μmol/L) demonstrated a stronger antagonistic effect on ZEA-induced cytotoxicity in this study, experiments with CUR alone showed a significant reduction in IPEC-J2 cell viability at concentrations ≥15 μmol/L. Such a biphasic dose–response pattern aligns with earlier studies, indicating that elevated concentrations of CUR can exert pro-oxidative or cytotoxic effects [50,51]. At elevated doses, CUR can negatively impact cells via several mechanisms: it may reduce mitochondrial membrane potential and damage the respiratory chain, leading to excessive reactive oxygen species generation [52,53]; it may interact with membrane lipids, compromising the bilayer’s structural integrity [54]; and it may partially disrupt key signaling pathways involved in cell proliferation and survival [55], culminating in potential cytotoxicity. Therefore, although CUR exhibits significant efficacy in alleviating ZEA-induced ferroptosis, its dosage should be carefully optimized in application to avoid cytotoxicity resulting from excessive use. Ming et al. reported that CUR interacts with p53, SLC7A11, and GPX4. While CUR increased p53 levels, it reduced both mRNA and protein levels of SLC7A11 and GPX4. CUR may induce ferroptosis by modulating the p53 and SLC7A11/GSH/GPX4 axis, thereby exerting inhibitory effects on colorectal cancer [20], which aligns with the molecular mechanisms revealed in this study following CUR treatment of IPEC-J2 cells. In another study, CUR encapsulated within yeast microcapsules (YMs) to form CM@YM was shown to protect Cur-MPN effectively and safeguard the gastrointestinal environment. CM@YM enhanced targeting and retention in inflammatory colons, and oral administration reduced ROS, decreased proinflammatory cytokines, and modulated macrophage polarization to M1, thereby restoring barrier function and maintaining intestinal homeostasis [56]. In summary, CUR alleviates ZEA-induced ferroptosis effectively by restoring iron homeostasis and the antioxidant barrier. However, despite CUR demonstrating excellent cytoprotective effects, its in vivo application still faces a significant challenge: its low solubility and bioavailability. Curcumin, being a natural polyphenolic compound, has low solubility in vivo, which significantly restricts its absorption in aqueous media. The poor water solubility of curcumin means that only a small amount is absorbed through the digestive tract when taken orally, resulting in a low plasma concentration. Current research indicates that curcumin’s bioavailability is generally lower than 1%, limiting its clinical therapeutic efficacy [57]. Consequently, the use of a cell model to evaluate the effects of CUR in this study may not fully represent its biological effects in vivo, particularly under the complex pharmacokinetic conditions of the body. In response to the low solubility and bioavailability of curcumin, recent research has introduced several potential solutions. On one hand, researchers have explored using nanotechnology to improve curcumin’s solubility and bioavailability [58]. Nanoparticles can increase curcumin’s solubility and improve its absorption in the body by enhancing drug delivery systems. On the other hand, curcumin can be effectively stabilized and its release rate enhanced by combining it with other drug carriers, such as liposomes, micelles, or solid lipid nanoparticles [59]. These strategies can not only increase the concentration of curcumin in the body but also extend the duration of its therapeutic effect. Therefore, although this study demonstrates the potential protective effects of curcumin against ZEA-induced ferroptosis in cell models, further animal and clinical trials are required to assess its efficacy and safety in clinical practice.

Apart from molecular alterations, mitochondrial structural disruption represents a hallmark morphological characteristic of ferroptosis. Serving as the central organelle for iron metabolism, decomposition, and synthesis, mitochondria are pivotal in maintaining iron homeostasis and orchestrating energy and material metabolism within cells [60]. Evidence suggests that mitochondrial injury and functional impairment facilitate oxidative stress, thereby triggering ferroptosis [61]. The primary distinction between ferroptosis and other forms of cell death lies in the structural alterations in mitochondria during ferroptosis. Mitochondrial morphological changes serve as an important parameter for assessing cell survival or death [62]. In previous studies on mouse embryonic fibroblasts (3T3-Swiss albino), mitochondrial membrane rupture was observed during ferroptosis induced by the GPX4 inhibitor RSL3, with the extent of rupture being time-dependent [63]. Our results showed that in IPEC-J2 cells exposed to ZEA, mitochondrial morphology exhibited marked alterations, including increased membrane density, and a substantial reduction in or complete loss of cristae, as well as shrinkage, deformation, and vacuolization. Damage to mitochondrial cristae and membranes is a typical morphological hallmark of ferroptosis, which is consistent with our findings. Nevertheless, administration of both low and high doses of CUR ameliorated the ZEA-induced mitochondrial damage to varying degrees. Therefore, this study further corroborates the protective role of CUR against ferroptosis from a morphological perspective.

In addition to preserving mitochondrial structural integrity, CUR’s protective effects may also originate from its interactions with the plasma membrane and associated receptors. Given its amphiphilic nature, CUR can incorporate into lipid bilayers, stabilizing membrane microdomains and influencing the spatial organization of receptor complexes [64]. Such modulation may alter the activity of redox-sensitive receptors and transporters, including SLC7A11, thereby enhancing cystine uptake and promoting glutathione synthesis [65,66]. These upstream effects could attenuate p53 activation and restore GPX4 expression, ultimately reducing ROS accumulation and lipid peroxidation [67,68]. This receptor–membrane interaction perspective provides a mechanistic link between CUR’s physicochemical properties and its ability to modulate the p53/SLC7A11/GPX4 signaling axis, further reinforcing its role in ferroptosis inhibition.

## 5. Conclusions

This study confirms that CUR effectively alleviates ZEA-induced ferroptosis in IPEC-J2 cells in vitro. CUR treatment not only suppressed the accumulation of Fe^2+^ and lipid peroxides but also restored glutathione levels, reduced ROS production, and preserved mitochondrial ultrastructure. Molecular analysis revealed that these protective effects were associated with the modulation of the p53/SLC7A11/GPX4 signaling pathway, reversing the ZEA-induced downregulation of SLC7A11 and GPX4 and the upregulation of p53 and ACSL4. These findings enrich the mechanistic understanding of ZEA toxicity and support the potential of CUR as a natural anti-ferroptotic agent. However, as this work was conducted in vitro, future studies should validate these results in vivo and explore strategies to improve CUR’s bioavailability, such as novel formulations or delivery systems, to facilitate its practical application in animal production and food safety.

## Figures and Tables

**Table 1:** Primer sequence list.

Gene	Primer Sequence (5′-3′)
ACSL4	Forward: 5′-CTGGAAATGCTGGGTTTGGGA-3′ Reverse: 5′-AGAGCCCGCCACACAAGTTA-3′
GPX4	Forward: 5′-TGTGTGAATGGGGACGATGC-3′ Reverse: 5′-CTTCACCACACAGCCGTTCT-3′
SLC7A11	Forward: 5′-CATCGGGACCATCATCGGAG-3′ Reverse: 5′-CATACGGTCCAGACGACCAG-3′
FTH1	Forward: 5′-ATTTGCGCTGCACGTGGT-3′ Reverse: 5′-GGGGGTCATTTTTGTCAGTGG-3′
p53	Forward: 5′-ACGCTTCGAGATGTTCCGAG-3′ Reverse: 5′-TTTTATGGCGGGAGGGAGAC-3′

## Data Availability

The data presented in this study are available in this article.
